# Antipsychotic Monitoring Within the Home Treatment Team in the Southern Trust, a Quality Improvement Project

**DOI:** 10.1192/bjo.2022.270

**Published:** 2022-06-20

**Authors:** Cedar Andress, Paul Coulter, Leah Watson

**Affiliations:** Southern Trust, Northern Ireland, United Kingdom

## Abstract

**Aims:**

The Royal College of Psychiatrists has a specialist group called the Home Treatment Accreditation Scheme (HTAS) that has published a set of best practice recommendations for Home Treatment Crisis Response (HTCR) teams across the UK. As of yet, the HTCR team in the Southern Trust is not accredited. We decided to focus our project on antipsychotic monitoring. SMART aim: All patients (100%) within the HTCR team commenced on antipsychotics are receiving an appropriate level of blood and physical monitoring as recommended by guidelines and these are being documented correctly within 10 days of discharge.

**Methods:**

PLAN

HTAS standards were reviewed alongside NICE guidelines on antipsychotic monitoring and a pro forma created. We collected baseline data on patients commenced on treatment dose antipsychotics in the HTCR team and assessed completion of bloods/ECGs/physical parameters and documentation.

DO

Our intervention for PSDA cycle 1 was to educate members of the multi- disciplinary team (MDT) via a presentation after the baseline data were analysed. We looked at correct documentation and how to fix common mistakes identified. We asked staff for their input on how to improve outcomes. Posters were printed off for guidance. We collected data after this intervention using the same pro forma.

STUDY

We analysed the results from PSDA cycle 1, comparing them to baseline results.

ACT

Our next step in PDSA cycle 2 would be to focus on continuing to improve poorer results such as prolactin levels and ECGs, with input from the MDT.

**Results:**

Baseline data showed between a 14% and 59% completion rate for various baseline bloods, 68–72% completion rate for heart rate (HR)/blood pressure (BP)/weight and a 36% completion rate for ECGs.

Following PDSA cycle 1, this improved to between a 55–100% completion rate for baseline bloods, a 91% completion rate for HR/BP/weight and a 64% completion rate for ECGs.

Baseline documentation of these parameters was correctly recorded between 9–68% of the time. This overall improved after PSDA cycle 1 to 18–73%.

**Conclusion:**

Our intervention from PDSA cycle 1 improved completion of bloods, physical parameters and ECGs in the HTCR team. Documentation also improved in all domains.

Our next step in PDSA cycle 2 would be to focus on continuing to improve poorer results, looking at altering practicalities that may have affected those areas.

